# Analysis of Peptide Ligand Specificity of Different Insect Adipokinetic Hormone Receptors

**DOI:** 10.3390/ijms19020542

**Published:** 2018-02-11

**Authors:** Elisabeth Marchal, Sam Schellens, Emilie Monjon, Evert Bruyninckx, Heather G. Marco, Gerd Gäde, Jozef Vanden Broeck, Heleen Verlinden

**Affiliations:** 1 Molecular Developmental Physiology and Signal Transduction, KU Leuven, Naamsestraat 59, P.O. Box 02465, B-3000 Leuven, Belgium; elisabeth.marchal@kuleuven.be (E.M.); sam.schellens@student.kuleuven.be (S.S.); emilie.monjon@kuleuven.be (E.M.); evert.bruyninckx@kuleuven.be (E.B.); jozef.vandenbroeck@kuleuven.be (J.V.B.); 2 Department of Biological Sciences, University of Cape Town, Private Bag, Rondebosch ZA-7700, South Africa; heather.marco@uct.ac.za (H.G.M.); gerd.gade@uct.ac.za (G.G.)

**Keywords:** AKH, carbohydrate, energy, GPCR, lipid, metabolism, neuropeptide, pest control

## Abstract

Adipokinetic hormone (AKH) is a highly researched insect neuropeptide that induces the mobilization of carbohydrates and lipids from the fat body at times of high physical activity, such as flight and locomotion. As a naturally occurring ligand, AKH has undergone quite a number of amino acid changes throughout evolution, and in some insect species multiple AKHs are present. AKH acts by binding to a rhodopsin-like G protein-coupled receptor, which is related to the vertebrate gonadotropin-releasing hormone receptors. In the current study, we have cloned AKH receptors (AKHRs) from seven different species, covering a wide phylogenetic range of insect orders: the fruit fly, *Drosophila melanogaster*, and the yellow fever mosquito, *Aedes aegypti* (Diptera)*;* the red flour beetle, *Tribolium castaneum*, and the large pine weevil, *Hylobius abietis* (Coleoptera); the honeybee, *Apis mellifera* (Hymenoptera); the pea aphid, *Acyrthosiphon pisum* (Hemiptera); and the desert locust, *Schistocerca gregaria* (Orthoptera). The agonistic activity of different insect AKHs, including the respective endogenous AKHs, at these receptors was tested with a bioluminescence-based assay in Chinese hamster ovary cells. All receptors were activated by their endogenous ligand in the nanomolar range. Based on our data, we can refute the previously formulated hypothesis that a functional AKH signaling system is absent in the beneficial species, *Apis mellifera*. Furthermore, our data also suggest that some of the investigated AKH receptors, such as the mosquito AKHR, are more selective for the endogenous (conspecific) ligand, while others, such as the locust AKHR, are more promiscuous and can be activated by AKHs from many other insects. This information will be of high importance when further analyzing the potential use of AKHRs as targets for developing novel pest control agents.

## 1. Introduction

Adipokinetic hormone was first isolated from the corpus cardiacum (CC)—a paired endocrine gland situated near the brain—of the locusts, *Locusta migratoria* and *Schistocerca gregaria* [1]. The function of AKH in insects lies primarily in the mobilization of energy reserves, such as lipids and carbohydrates, from the insect fat body at times of high physical activity (such as flight and locomotion); AKH is also believed to play a role in egg production, larval development, immune and stress responses [2,3,4,5,6,7,8,9,10]. Furthermore, other reports have stated a functional role for AKH in stimulating locomotor activity, heart rate and other myotropic contractions, and in inhibiting protein and lipid synthesis in diverse insect species [3,11,12,13]. In the molecular genetic model organism, the fruit fly *Drosophila melanogaster*, AKH has been implicated in nutritional and oxidative stress responses and in extending lifespan under starvation conditions [14,15,16,17]. Since its first identification, the presence of AKH has been determined not only in many different insect species, but recently also in other members of the Ecdysozoa, as well as in Lophotrochozoa [18].

All AKHs are derived from precursor proteins and are preceded by a secretory signal peptide and followed by an AKH precursor-related peptide, for which the function is currently still unknown [16]. Some insects, such as the locusts, *L. migratoria* and *S. gregaria*, have multiple AKH precursor genes encoded in their genome, resulting in the presence of multiple mature AKHs in their CC [19]. The structure of AKHs was recently reviewed by Li et al. [18], stating that true AKHs compose a family of neuropeptides 8 to 10 amino acids in length, typically having a pyroGlu (pQ) group at their N-terminus (position 1); an aliphatic or aromatic amino acid residue at position 2; Phe-Ser (FS), Phe-Thr (FT) or Tyr-Ser (YS) residues in positions 4 and 5; and either a Trp-amide (Wa), Trp-Gly-amide (WGa) or Trp-Gly-Xxx-amide (WGXa) at their C-terminus (position 8 and/or following). AKHs closely resemble the red pigment concentrating hormones (RPCH) of Crustacea; in fact, Panbo-RPCH is synthesized in decapod crustaceans, as well as in a number of insect orders [20] hence they are grouped as the AKH/RPCH family of peptides, which are considered arthropod homologues of the vertebrate gonadotropin-releasing hormone (GnRH) peptide family [21,22,23].

During energy-demanding processes, AKH will be released from the CC into the hemolymph, and will interact with its receptor present in the membrane of the fat body adipocytes, inducing the release of energy-rich substrates, such as lipids, trehalose or proline [24]. In 1998, Hauser et al. [25] cloned a *D. melanogaster* G protein-coupled receptor related to vertebrate GnRH receptors, and later it was found to be the AKH receptor [26,27]. Since then, several additional AKH receptors were unequivocally pharmacologically identified in insects: the cockroach, *Periplaneta americana* [28,29]; in the mosquito, *Anopheles gambiae* [30]; in the silkworm, *Bombyx mori* [27,31,32], the fleshfly, *Sarcophaga crassipalpis* [33], the tsetse fly, *Glossina morsitans* [34] and in the beetle, *Tribolium castaneum* [35]. Very recently, Li et al. [18] characterized two AKH receptor splice variants in the oyster, *Crassostrea gigas*, thereby proving the functionality of this signaling system in mollusks and tracing its evolutionary origin back to more than 550 million years ago.

AKH receptors are also closely related to the corazonin (Crz) and the AKH/Crz-related peptide (ACP) receptors, all belonging to the GnRH receptor superfamily [8,18]. The ACP receptor is activated by a neuropeptide very closely related to both AKH and Crz, and was therefore named ACP [35]. ACP and its receptor are structural intermediates between the AKH and Crz peptides and receptors, respectively, and therefore represent a good example of receptor–ligand co-evolution [8,18]. 

In the genome of the beneficial insect species, *Apis mellifera*, an AKH receptor ortholog was identified [36] but in several studies it was suggested that a functional AKH signaling system would be absent because of the inability to detect an *A. mellifera* AKH using mass spectrometry methods [8,19]. Veenstra et al. [19] linked this to the presence of a second TATA box in the promoter region of the gene thought to prevent AKH release. However, other studies could detect changes in AKH receptor expression upon immune or heat challenges [37,38,39] hinting that the receptor does play a role in physiological processes. Moreover, a recent study by Sturm et al. [40] has reported the AKH signal in the glandular part of the *A. mellifera* CC. This prompted further investigation. Hence, in the current study we aim to test the functionality of the recombinant receptor in an in vitro calcium assay together with several other AKH receptor orthologues amplified from different insect orders.

In our study we compare the sensitivity of AKH receptors to a selection of naturally occurring AKHs, as well as the related neuropeptides ACP and Crz. The AKH receptors of the following insects are investigated: the fruit fly, *D. melanogaster*; the yellow fever mosquito, *Aedes aegypti*; the red flour beetle, *T. castaneum*; the pine weevil, *Hylobius abietis*; the honeybee, *A. mellifera*; the pea aphid, *Acyrthosiphon pisum*; and the desert locust, *S. gregaria*. Our results show that the AKH receptor of *S. gregaria* is the least selective of the tested receptors, whereas the AKH receptors of the evolutionarily and ecologically more specialized dipteran species, *A. aegypti* and *D. melanogaster*, are less tolerant to amino acid changes in the peptide. As AKH is a pleiotropic hormone controlling a variety of physiological processes in insects, information from our study may be useful in the design of a new generation of bio-rational peptide-based agents for pest management.

## 2. Results and Discussion

### 2.1. Cloning of the Receptors

We selected seven insects based on availability of AKH receptor (AKHR) sequence data and the relative importance of the insects to human society, viz. well-known pest species in agriculture, forestry and stored foods, disease vectors, and a beneficial pollinator species. The selected species span a range of insect orders. The AKHR cDNA sequences of these insects were cloned. Five of the receptor sequences were available online via BLAST search of the NCBI database: *D. melanogaster* AKHR (GenBank acc. no. NP_477387), *A. aegypti* AKHR (GenBank acc. no. CAY77166), *A. pisum* AKHR (GenBank acc. no. XP_003245941), *A. mellifera* AKHR (GenBank acc. no. NP_001035354) and *T. castaneum* AKHR (GenBank acc. no. NP_001280549). For *A. aegypti*, two very similar sequences were found (Genbank acc. No. CAY77164 and CAY77166). Both could be amplified and cloned and were functionally active when expressed. Since we did not detect any differences in the activity and selectivity of these two receptor variants and since both receptors have been described in the past [41], further analyses were done with just one (GenBank acc. no. CAY77166). The complete sequence of an AKHR was found in the in-house transcriptome database of *S. gregaria* (GenBank acc. no. MG544188). Prof. Julian Dow (University of Glasgow, Glasgow, UK) kindly provided partial sequences of a possible AKHR of *H. abietis*, derived from an in-house transcriptome. Based on that sequence we amplified and cloned a complete, functional AKHR (GenBank acc. no. MG562511). The receptor sequences, as verified by Sanger sequencing of the cloned insect cDNAs, are shown in a multiple alignment in Supplementary Figure S1. All receptors contain the conserved amino acids that are typical for rhodopsin-like GPCRs and for the AKHR/RPCHR/GnRHR receptor subfamily, as indicated in the supplementary figure.

### 2.2. Phylogenetic Tree

**Figure 1:**
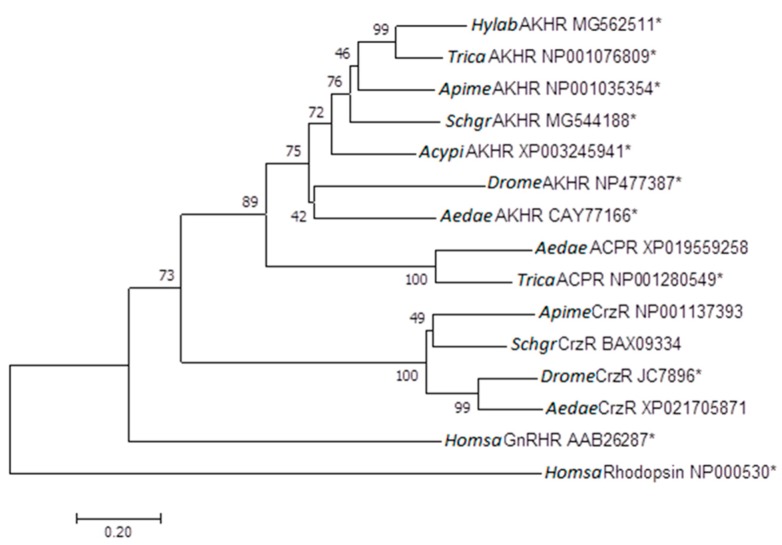
Phylogenetic analysis of the gonadotropin-releasing hormone (GnRH) receptor subfamily (i.e., GnRH/AKH/RPCH/ACP/Crz-type receptors) was inferred using the neighbour-joining method [44]. Phylogenetic analysis was conducted by using MEGA version 7 [45]. Alignments were conducted with multiple sequence comparison by log-expectation (MUSCLE). Bootstrap-support percentages are based on 1000 replicates using the Jones Taylor Thornton model and are indicated on the nodes [46]. The human rhodopsin receptor was used as an outgroup to root the tree. Proteins marked with an asterisk ‘*’were pharmacologically characterized. The scale bar allows conversion of branch length in the dendrogram to genetic distance between clades (0.2 = 20% genetic distance). Abbreviations used: AKHR, adipokinetic hormone receptor; ACPR, AKH/Crz-related peptide receptor; CrzR, corazonin receptor; GnRHR, GnRH receptor.

The analyzed insect AKH receptors form a separate cluster and are distinct from ACP, Crz and GnRH receptors (Figure 1). This phylogeny is well in line with what other studies have described [11,35,42,43].

**Figure 2:**
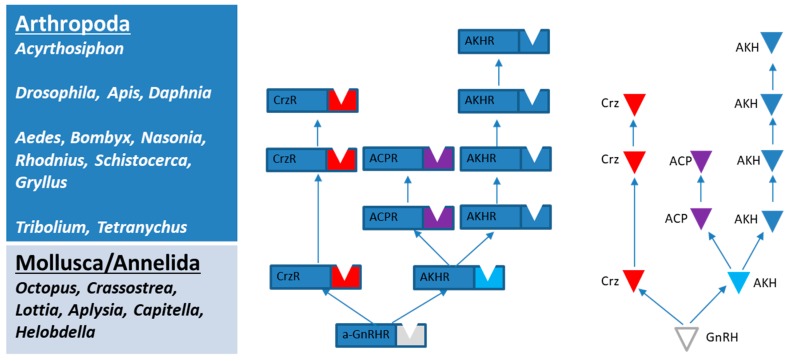
Presence of the AKH/ACP/Crz signaling systems in different arthropod species, based on present study and literature data [11,35,49].

We did not find any ACP or Crz receptors, nor the corresponding peptide precursors, in the genome of *A. pisum*, which suggests that some insect species lack the related Crz and ACP signaling systems and only have the AKH system (Figure 2) (see [47]). Similarly, other receptor–peptide couples have disappeared in some insect species, which is the case for, e.g., allatotropin signaling in *D. melanogaster* [48] and sulfakinin signaling in *A. pisum* [8].

### 2.3. Cell-Based Receptor Activity Assays

**Figure 3:**
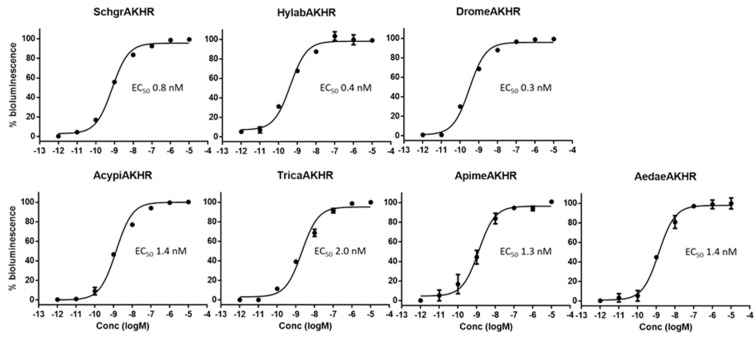
Dose–response curves of the endogenous AKHs with their respective cognate AKH receptors in vitro. The receptors were expressed in CHO cells and tested in an aequorin-based bioluminescent receptor assay. Data are shown as mean ± SD, each experiment was performed at least in duplicate, and % bioluminescence is a proxy for receptor activation. Values indicated on the *x*-axis refer to the logarithm of the ligand concentration (expressed in molar). Values indicated on the *y*-axis are the percentage relative to the maximal bioluminescence level obtained in the assay, where 100% was defined as the average value obtained when applying high doses (1 and 10 µM) of the respective AKHs and 0% was defined as the response measured when no peptide was applied (BSA control). Table 1 shows the amino acid sequences of the tested peptides.

We expressed the different insect AKH receptors in Chinese hamster ovary (CHO) WTA11 cells, stably expressing apo-aequorin, a zeocin resistance gene and the promiscuous Gα_16_ subunit coupling to the phospholipase C and Ca^2+^ signaling cascade (Euroscreen, Brussels, Belgium). The AKH from each species elicited a clear dose-dependent response in the bioluminescent calcium assay of its conspecific receptor, with an EC_50_ value in the low nanomolar range (Figure 3).

Our data show for the first time that honeybees do have a functional AKH receptor that responds in a dose-dependent manner to an AKH that had been predicted from the *A. mellifera* genome [50,51] and was detected by mass spectrometry by Sturm et al. [40]. Together with other studies that showed changes in AKH receptor expression upon immune challenges and temperature stress [37,38,39], our data strongly suggest that honeybees have a functional AKH system [19,40].

**Figure 4:**
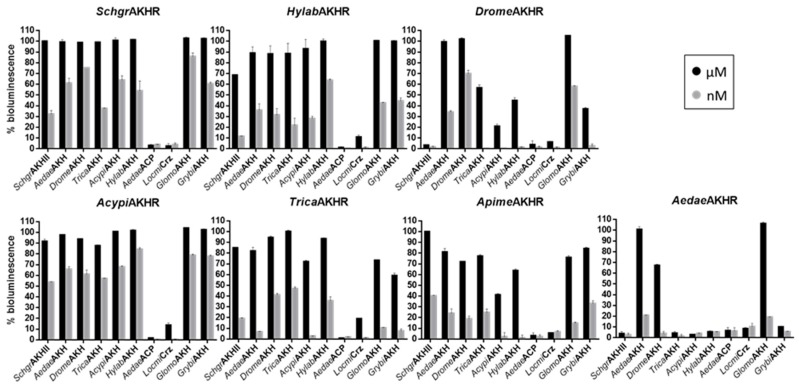
Receptor activation as assessed by a cell-based bioluminescent calcium assay: different insect peptides (indicated on the *x*-axis) belonging to the AKH/RPCH, ACP and Crz peptide families were tested at micromolar (1 µM) and nanomolar (1 nM) concentrations on seven different insect AKH receptors. Table 1 shows the amino acid sequences of the tested peptides. The *y*-axis indicates the percentage relative to the maximal bioluminescence level obtained in the assay + SEM, where 100% was defined as the average value obtained when applying high doses (1 and 10 µM) of the respective cognate AKH and 0% was defined as the response measured when no peptide was applied (BSA control).

In addition, AKH peptides derived from other insect species (Table 1) activated several heterospecific AKH receptors, although much higher concentrations were required in some cases (Figure 4). The most conserved AKH residues for receptor activation are Phe4 and Trp8. Other studies have indicated that replacement of these residues abolishes activation at previously analyzed AKH/RPCH receptors [34,52,53]. The importance of the residues in these positions is also evidenced by the alignments and sequence logos provided for AKH in DINeR, a Database for Insect Neuropeptide Research [54]. These residues are present in the three tested peptide families (AKH/RPCH, ACP, Crz), although in Crz they are separated by five amino acid residues instead of three, which can explain why Crz is not able to activate any of the AKHRs that were analyzed to same extent as the endogenous AKHs at micromolar concentrations. 

The main differences between these two tested peptides lie in the presence of the very hydrophilic Arg-Asp (RD) residues at positions 5 and 6 in ACP versus a hydrophobic Pro and a neutral Thr (PT) in *Acypi*AKH, as well as the combination of a neutral Asn and hydrophobic Ala (NA) at positions 9 and 10 of ACP, versus the hydrophobic Gly and neutral Gln (GQ) of *Acypi*AKH (Table 1), which most likely prevents the molecules from having the same conformation. ACP is structurally more related to AKH than to Crz, but only very low levels of AKHR activation were observed with ACP, even when applied in micromolar concentrations. This may be the result of a selective pressure against cross-activation between the AKH and ACP signaling systems, which often co-exist in insects (see Figure 2). The mollusk *C. gigas* does not have an ACP system and ACPs from insects were almost equally potent as the endogenous ligand at activating the *C. gigas* AKH receptor [18]. Also, no co-activation of the ACP and AKH systems was observed in *T. castaneum* [35]. Also in the kissing bug *Rhodnius prolixus*, and the mosquito *A. gambiae*, AKH, Crz and ACP receptors are only activated by their corresponding cognate ligand [35,55]. In addition, we only detected a very limited activation of the AKHRs from beetles (*T. castaneum* and *H. abietis*) and aphids (*A. pisum*) with Crz at micromolar concentrations (Figure 3). Remarkably, these species do not have an endogenous Crz signaling system. Perhaps, this may mean that the evolutionary selection against cross-activation between the Crz and AKH peptide–receptor couples was discontinued after the loss of the Crz system in their respective ancestors.

The AKH receptors of the two dipteran species that we tested appear to be the most selective towards their endogenous ligand. Only the AKHs from other dipterans could elicit a response in the nanomolar range (Figure 3). The primary structures of these peptides are very similar (pQLTFXPXWamide), only differing at the more permissive positions 5 and 7 (Table 1). These observations are in line with previous structure-activity analyses on the AKH receptors of *D. melanogaster*, *A. gambiae* [52] and *G. morsitans* [34]. The tested dipteran AKH receptors can best cope with a change at position 7 of the peptide. At position 5, a Thr or Ser residue is typically present, while its replacement to Gly almost completely abolished peptide-mediated receptor activation [34,52]. The reduced dipteran AKHR activation that is observed with the other tested peptides is likely caused by amino acid changes at positions 2 or 3 since replacing Thr3 to Asn3 almost completely abolished peptide-induced activation of the *G. morsitans* receptor [34].

Some insects have more than one AKH precursor gene, and consequently have more than one AKH peptide. For example, *S. gregaria* has three AKH peptides. *Schgr*AKH-I (present also in the migratory locust) is a decapeptide, *Schgr*AKH-II (which is also present in *A. mellifera*) and *Schgr*AKH-III (which is identical to *Aedae*AKH). However, only one locust AKH receptor gene is currently known. This situation may explain why this locust receptor is more permissive in accepting amino acid changes without displaying a severely altered activation. The question remains why there might be a need for multiple endogenous peptides of a single receptor type in a particular species. At present, the answers to this question are still unclear, but this situation is not exceptional as there are many other examples where multiple natural peptides are shown to act as agonists for the same receptor [56,57]. These peptides might perhaps be redundant. However, it is also possible that the presence of multiple AKH precursor genes allows for a more specific control of the expression, storage and release of the respective AKH peptides. Also, the different peptides may have a different stability and bioavailability at different body locations. In addition to the AKHs, there may also be a function for the associated peptides encoded in these AKH precursors. Moreover, it cannot be excluded that the structural and functional properties of the AKHR itself may be fine-tuned in vivo, depending on the concrete cellular context in which it is expressed, and for which the presence of different endogenous ligands may have biological relevance. Furthermore, it will always remain difficult to fully prove the complete absence of any other, currently unknown, receptor type.

The development of resistance against existing insecticides and problems related to their unspecific working mechanism underline the necessity for research focused on the design of new, biorational insecticidal compounds with a more selective mode of action. The possible use of neuropeptide-receptor based control strategies has been proposed in the past. The rational design of peptide analogues will allow for the selectivity that is often problematic when applying the currently commercially available insecticides [58,59,60]. Since AKH is a peptide regulating different crucial physiological processes in insects, the development of AKH-based insect control agents may be part of the solution by offering several advantages, such as being less toxic, more specific and less prone to degradation than regularly used commercial chemicals. Our results can clearly contribute to the development of AKH analogues that target specific insect populations rather than other organisms. In future, our in vitro tests will have to be correlated to in vivo tests to verify the effects of rationally designed peptide analogues on the fitness of the targeted insect populations. 

## 3. Materials and Methods

### 3.1. Molecular Cloning

RNA was extracted from adult whole animal *H. abietis*, *A. mellifera*, *A. gambiae*, *A. pisum*, *T. castaneum* and *D. melanogaster* using the RNeasy Lipid Tissue Kit (Qiagen, Hilden, Germany) following the manufacturer’s protocol. Fat body from adult male *S. gregaria* was dissected and RNA was extracted as described above. RNA was reverse transcribed using Roche’s Transcriptor High Fidelity cDNA Synthesis Kit as per manufacturer’s recommendation. The resulting cDNA was used in this study to amplify the adipokinetic hormone receptor transcripts of the specified insects using the primers listed in Table 2.

We cloned the resulting PCR product into pcDNA3.1/V5-His-Topo directional expression vector and transformed to One Shot^®^ TOPO10 chemical competent *Escherichia coli* cells according to the manufacturer’s guidelines (Invitrogen, Carlsbad, CA, USA). The cells were grown overnight at 37 °C on Luria Bertani (LB) agar plates (35 g/L; Sigma-Aldrich, St. Louis, MO, USA) containing 10 mg/mL ampicillin (Invitrogen). We transferred grown colonies to 5 mL LB medium (with 10 mg/mL ampicillin; Sigma-Aldrich). After growing overnight at 37 °C, the receptor DNA containing vector was purified using ‘GenElute™ HP Plasmid Miniprep’ kit (Sigma-Aldrich) and sequenced using the ABI PRISM 3130 Genetic Analyzer (Applied Biosystems, Foster City, CA, USA). Colonies containing the correct vector were used to inoculate 100 mL LB medium with 10 mg/mL ampicillin and were grown overnight at 37 °C in a shaking incubator. We purified the plasmids using the ‘GenElute™ Plasmid Maxiprep Kit’ (Sigma-Aldrich).

### 3.2. Phylogenetic Analyses

We compared the amino acid sequences of the AKH receptors tested in this study with other members of the AKH/ACP/Crz/GnRH receptor family. We aligned the amino acid sequences of the selection of receptors by using multiple sequence comparison by log-expectation (MUSCLE). In addition, we constructed a phylogenetic tree of a selection of receptors with the neighbour-joining method (1000-fold bootstrap resampling) using the Jones Taylor Thornton mutation data matrix. We performed all analyses with the MEGA software version 7 [45].

### 3.3. Cell Culturing and Transfection

We analyzed the activity of the different AKH receptors using CHO-WTA11 cells, which contain a promiscuous G_α16_ subunit that will induce an intracellular Ca^2+^-increase upon receptor binding independent of the receptors’ natural signaling cascade (see also [48]). We cultured the cells at 37 °C with constant supply of 5% CO_2_ in Dulbecco’s Modified Eagle’s Medium Nutrient Mixture F-12 Ham (DMEM/F12) with l-glutamine, 15 mM HEPES, sodium bicarbonate and phenol red (Sigma-Aldrich) enriched with 10% heat-inactivated fetal bovine serum (Gibco), 100 IU/mL penicillin and 100 μg/mL streptomycin (Gibco) and 250 mg/mL Zeocin (Gibco).

For transfection of the cells (T75 flasks at 60–80% confluency), we dissolved 5 µg pcDNA3.1-receptor or empty pcDNA3.1 vector in 2.5 mL Opti-MEM^®^ (Gibco) supplemented with 12.5 µL Plus™ Reagent of the Lipofectamine LTX Kit (Invitrogen) in 5 mL polystyrene round-bottom tubes. We incubated this mixture for 5 min at room temperature. Thereafter, we added 30 µL LTX and incubated this mixture again at room temperature for 30 min. We then removed the cell medium and added the transfection mixture dropwise followed by 3 mL fresh complete culture medium. After an overnight incubation at 37 °C with constant supply of 5% CO_2_, we added 10 ml of complete culture medium and allowed the cells to grow for another night (37 °C, 5% CO_2_). We monitored ligand-induced changes in intracellular Ca^2+^ in the cells as described below.

### 3.4. Calcium Reporter Assay

We detached the transfected cells using phosphate buffered saline (PBS), supplemented with 0.2% EDTA, and rinsed them of the flask with DMEM/F12 with l-glutamine and 15 mM HEPES (Sigma-Aldrich). We determined the number of viable and nonviable cells with the TC20 automated Cell Counter (Bio-Rad, Hercules, CA, USA). In order to achieve a cell density of 5 × 10^6^ cells/mL, we centrifuged the cells for 5 min at 800 rpm and resuspended them in the appropriate volume of sterile filtered bovine serum albumin (BSA) medium (DMEM/F12 with l-glutamine and 15 mM HEPES, complemented with 0.1% BSA). In addition, we loaded the cells with 5 µM Coelenterazine_h (Invitrogen) by gently shaking them at room temperature for 4 h in the dark to reconstitute the holo-enzyme aequorin. Before exposure to potential ligands dissolved in BSA medium, we diluted the cells tenfold in the same medium 30 min prior to the measurement. Thereafter, the Mithras LB 940 (Berthold Technologies, Bad Wildbad, Germany) injected 50 µL of the cells into every well (25,000 cells/well) of a 96-well plate. The machine measured the ligand-induced calcium response for 30 s, whereafter it added 50 µL of 0.1% Triton X-100 in order to measure the total cellular Ca^2+^-response. The ligand-specific response was normalized using the total response (ligand + Triton X-100), which is directly related to the number of cells present in the well. A negative control (only BSA) was included in each row to correct the cell response of each well of the same row. We performed the calculations using the output file from the Microwin software (Berthold Technologies) in Excel (Microsoft). Further analysis was done in GraphPad Prism 6.

### 3.5. Synthetic Peptides

The AKH peptides were custom-synthesised by Pepmic Co., Ltd. (Suzhou, China) or by Synpeptide Co. (Shanghai, China).

## Figures and Tables

**Table 1:** Primary structure of the peptides used in the current study. The conserved residues are shown in bold, while dashes were introduced to align the sequences. Abbreviations used: AKH, adipokinetic hormone; Crz, corazonin; ACP, AKH/Crz-related peptide.

Neuropeptide Name	Amino Acid Sequence
*Aedae*AKH = *Schgr*AKH-III	**pQ**LT**F**--TPS**W**--**amide**
*Drome*AKH	**pQ**LT**F**--SPD**W**--**amide**
*Glomo*AKH	**pQ**LT**F**--SPG**W**--**amide**
*Trica*AKH	**pQ**LN**F**--STD**W**--**amide**
*Hylab*AKH *= PeramCAH-I*	**pQ**VN**F**--SPN**W**--**amide**
*Grybi*AKH	**pQ**VN**F**--STG**W**--**amide**
*Schgr*AKH-II = *Apime*AKH	**pQ**LN**F**--STG**W**--**amide**
*Acypi*AKH	**pQ**VN**F**--TPT**W**GQ**amide**
*Aedae*ACP	**pQ**VT**F**--SRD**W**NA**amide**
*Locmi*Crz	**pQ**-T**F**QYSHG**W**TN**amide**

**Table 2:** Sequences of the primers used in this study to pick-up the adipokinetic hormone receptors of the specified insects. Note that we added a CACC sequence in front of each forward primer to obtain a kozak sequence necessary for expression in vertebrate cell lines. Primer specific annealing temperatures (Tm: melting temperature) are indicated in degrees Celsius.

Species	Forward Primer	Reverse Primer	Tm
*A. pisum*	CACCATGGAAGTGATGGATTCTGACGCC	GTTAGTTCACAAATTGTACCAGATTACC	63
*A. mellifera*	CACCATGGAAAGCAGTATAAAAATAATCACC	TTAATCAAGAAGTTTGAGCGATAATGATAATGG	66
*A. aegypti*	CACCATGTCAAATGCAATTTTGAAAACAG	TCAGACTGGTTTGATTGCACAT	61
*D. melanogaster*	CACCATGGCAAAAGTAGCTGAG	TTACTTCTGGCGGATCGG	64
*H. abietis*	CACCATGAAGGAACTAAAAGATTCCCC	TCATTTTTCGATGTTGTTTCTTTGTAA	60
*S. gregaria*	CACCATGGCGGGCCTCGAATCGG	TCACCTTGCCTCCGTTGTTCTG	59
*T. castaneum*	CACCATGAACTTTAGTGAGACTCTTTGGA	CTATTCTAAAGTCTTCAGTGATATCTCA	62

## Supplementary Materials

Supplementary materials can be found at http://www.mdpi.com/1422-0067/19/2/542/s1.
