# Supplementary material for: Analysis of Peptide Ligand Specificity of Different Insect Adipokinetic Hormone Receptors
Source: Int J Mol Sci. 2018 Feb 11;19(2):542. doi: 10.3390/ijms19020542 (PMC5855764; doi:10.3390/ijms19020542)
Supplement: Supplementary file 1 [file ijms-19-00542-s001.pdf]

```
DromeAKHR                                    MAKVAEENDHRDLSNWSNVN-----DTNGTIHLTKDMVFNDGHRLS  41
AedaeAKHR MSNAILKTERGEVLNYSHSYGENYNNDVNTMPYVLSSSTSKTGVFDNETWYGTN-----SSNWNEPLPIDMQFNDGHKLQ  75
AcypiAKHR                      MEVMDSDANT---VLHSVTKGVHVGPPPVWPGMDNVTNASMFDESNLPYDMKFNEGHVVA  57
SchgrAKHR                                          MAGLESENLWSQ---------SNNSVLPEDMKFNDGHVVS  31
ApimeAKHR                                     M-ESSIKIITTTGLENWRV--N----NSNYTELLPIDMRFNEGHIVS  40
HylabAKHR                          MKELKDS---P-DASETVQDHRILLDWSDTTN----ISDPEHHLPINMQFNDGHRLS  49
TricaAKHR                    MNFSETLWKMKDP---M-SASETVQDHRNLLDWSKTSL----DNATEHKLPISMRFNEGHQLS  55

                    TM1 •                     •   •  TM2          •            •
DromeAKHR ITVYSILFVISTIGNSTVLYLLTKRRLRGPLRIDIMLMHLAIADLMVTLLLMPMEIVWAWTVQWLSTDLMCRLMSFFRVF 121
AedaeAKHR IVVYSVLMVISAIGNITVLALLIKRRLKSHSRIDMMLTRLAIADLLVTFLMMPLEIGWAATVQWRAGDIMCRVMAFFRTF 155
AcypiAKHR IVTYSILMVVSAIGNITVLTIILKRRRKAGTRIHAMLMHLAIADLLVTFLMMPLEITWAWTVQWVLGDPLCRIMSFFRIF 137
SchgrAKHR IATYSVLMVVSAAGNLTVLFIILRR--RARSRVNHMLMHLAIADLLVTFLMMPLEIGWSSTVSWRAGDALCRVMAFFRVF 111
ApimeAKHR IVFYSVLMIISAIGNTTVLILITCRKRVSKSRIHIMLMHLAIADLLVTFLMMPLEIGWAITVSWKAGDVMCRIMAFFRMF 120
HylabAKHR ITVYSVLMVFSAVANITILVLLVKRRRQQPSRINTMLMHLAIADLLVTFLMMPLEIAWAFTVQWLAGDVMCRIMSFFRIF 129
TricaAKHR IIVYSILMVFSAIANTTVLVLIVKRRRKTPSRINTMLMHLAIADLLVTFLMMPLEIGWASTVSWYAGDAMCRIMMFFRMF 135

           •  TM3       ••      *                  TM4     •*    *              *•
DromeAKHR GLYLSSYVMVCISLDRYFAILKPLKRS--YNRGRIMLACAWLGSVVCSIPQAFLFHLEEHPAVTGYFQCVIFNSFRSDFD 199
AedaeAKHR GLHLSSFVLVCISVDRYYAVLQPLNLS--KSRGKIMILIAWAMATLCSAPQPFIFHVEIHPNHTWYEQCVTYNTFSNDNY 233
AcypiAKHR GLYLSSFILICISVDRYLAVLQPMRLYQMDRRGKLMIAVAWIASVVCSLPQTYIFHVERHPNATWYEQCVTYNAFSSKLH 217
SchgrAKHR GLFLSSFVLICISIDRYFAVLKPMKLSDVARRGRYLLAAAWIMSVLCSTPQMFVFHVESHPNVTWYRQCVSYNSFPSKLS 191
ApimeAKHR GLYLSSFVLVCISMDRYYAVIKPLQLWDVDKRGKIMLSFAWIGSVVCSLPQTIVFHLETHPNVTWYSQCVTFNAFPTYTH 200
HylabAKHR GLYLSSFVLCCISIDRYYAVLKPLHLVDLDRREKCLIISAWMGAVICSTPQMFLFHTENHPNITWYVQCVTYHSFPTYAQ 209
TricaAKHR GLYLSSFILVCISVDRFYAVLKPLYLRALDRRDKFMLLGAWLGATLCSIPQMVVFHVESHPNITWYQQCVTYNVFPTYAH 215

            *        TM5  •*                                                TM6  •      •
DromeAKHR EKLYQAASMCSMYAFPLIMFIYCYGAIYLEIYRKSQRVLKDVIAERFR-RSNDDVLSRAKKRTLKMTITIVIVFIICWTP 278
AedaeAKHR HTVYNILVMMFMYALPLLTIICSYASIYMEIFRHSRMPN----SEGFR-RSSIDALSRAKRRTLKMTITIVMAFVICWAP 308
AcypiAKHR ELAYLYFGMFMMYWLPLIVILFCYASIIIEIYRRSRESI---CGQGTDNVRRLGFLGRAKSRTLKMTIIIVIVFVVCWTP 294
SchgrAKHR EYSYLGFGMLMMYGLPLAVIIFSYTSILGEIYRRSREFS----DDRFR-RSSLGFLGRARIRTLKMTIIIVVVFFICWTP 267
ApimeAKHR EITYSLFGMIMMYWFPLVVIIYTYTSILLEIRRRSKKSE----DDKIR-RSSIGFLTRAKIRTLKMTVIIIAVFFICWTP 275
HylabAKHR ELAYSISGMVMMYAFPLGVIIFSYASILLEIFRRTRNPG---CADSVT-RSSLGFLGKAKIRTLKMTIIIVFVFFVCWTP 285
TricaAKHR ELTYLLFGMVMMYALPLAVIIFSYASILLEIRRRTRNPY----GDSVT-RSSLAFLGKAKVRTLKMTIIIVLVFFVCWTP 290

                                 TM7 •   •*
DromeAKHR YYTISMWYWLDKHSAGKINPLLRKALFIFASTNSCMNPLVYGLYNIRGRMNNNNPSVNNRHTSLSNRLDSSNQLMQKQLT 358
AedaeAKHR YYVMSVWYWLDQKSAENVDQRVQKGLFLFACTNSCMNPIVYGIYNVKLRKKKKPDGVKSGQSSVILRNSAK-----Y--T 381
AcypiAKHR YYIMAIWYWTDHKSAQMVDQKVQHALFMFACTNSCMNPIVYGAFNIRTRRTLVTQGVGES--------VASVRVVTW--H 364
SchgrAKHR YYIMCLWWWFDKDSAMKIDFRLQKGLFIFASTNSCMNPIVYGVFNIRARPDRGQKGKQQQVTSRSGRGVKVICLVSW--K 345
ApimeAKHR YYVMSLWYWIDRNSAYKIDQRIQKGLFLFACTNSCMNPIVYGAFNIRDRNKTSARP------------------------- 331
HylabAKHR YHVMCVWYWYDRDSALHVDQRIQKGLFLFACTNSCANPVVYGIFNIRARRKTAKVRPRLNS-------------NAI--R 350
TricaAKHR YYVMCIWYWLDRESAKNVDQRIQKALFLFACTNSCMNPVVYGVFNIRARRTGRKVSPRVNT-------------I----K 363

DromeAKHR NNSLLNGRGQVMAAAVSATTKLANVVSLKGTANGNGSAAAAGTVPITPPLTVTIAPL-ATDDEANDDSCLSAVTIRCQDQ 437
AedaeAKHR R---------------HSESIRSSSVWF                                                     394
AcypiAKHR KLTV-------RTKANRKSTIGNKAALFKSAQNGN--TNDDGLVKENVTVTTTLCNDSDINDKNT                 420
SchgrAKHR RPPK-------VPP--------------------------------GAADRPYVCSSCGATDRTTEAR              374
ApimeAKHR ---------------------------------------------TTIETRVTP-LSLSLKLLD                  349
HylabAKHR RPSF-------IPP--------------------------------SITSETRLSP-IEISLRSLE                376
TricaAKHR HTSC-------I-P--------------------------------TPNGDSRLPP-LEISLKTLE                388

DromeAKHR SPIRQK                                                                            443
```

Figure S1. Amino acid sequence alignment of the *D. melanogaster* AKHR (GenBank acc. no. NP_477387), *A. aegypti* AKHR (GenBank acc. no. CAY77166), *A. pisum* AKHR (GenBank acc. no. XP_003245941), *S. gregaria* AKHR (GenBank acc. no. MG544188), *A. mellifera* AKHR (GenBank acc. no. NP_001035354), *H. abietis* AKHR (GenBank acc. no. MG562511) and *T. castaneum* AKHR (GenBank acc. no. NP_001280549). The amino acid position is indicated on the right. Identical residues between all the receptors are highlighted in black and conservatively substituted residues in grey. Dashes indicate gaps that were introduced to maximize homologies. Putative transmembrane regions (TM1–TM7) are indicated by grey bars. Conserved amino acids for the rhodopsin-like GPCRs are indicated by a dot (•), whereas conserved amino acids for the GnRH receptor subfamily are indicated by asterisk.
